# Correlation Between Resting Theta Power and Cognitive Performance in Patients With Schizophrenia

**DOI:** 10.3389/fnhum.2022.853994

**Published:** 2022-04-22

**Authors:** Yanxiang Cao, Chuanliang Han, Xing Peng, Ziyao Su, Gan Liu, Yixi Xie, Yiting Zhang, Jun Liu, Pei Zhang, Wen Dong, Michel Gao, Sha Sha, Xixi Zhao

**Affiliations:** ^1^The National Clinical Research Center for Mental Disorders and Beijing Key Laboratory of Mental Disorders, Beijing Anding Hospital, Capital Medical University, Beijing, China; ^2^Advanced Innovation Center for Human Brain Protection, Capital Medical University, Beijing, China; ^3^Shenzhen Key Laboratory of Neuropsychiatric Modulation and Collaborative Innovation Center for Brain Science, Guangdong Provincial Key Laboratory of Brain Connectome and Behavior, CAS Center for Excellence in Brain Science and Intelligence Technology, Brain Cognition and Brain Disease Institute, Shenzhen Institute of Advanced Technology, Chinese Academy of Sciences, Shenzhen–Hong Kong Institute of Brain Science, Shenzhen Fundamental Research Institutions, Shenzhen, China; ^4^School of Public Health, North China University of Science and Technology, Tangshan, China; ^5^Beijing Pinggu Hospital-Beijing Friendship Hospital, Capital Medical University, Beijing, China; ^6^WM Therapeutics Ltd., Beijing, China

**Keywords:** schizophrenia, EEG, theta, cognitive performance, MCCB

## Abstract

**Objective:**

Schizophrenia is a mental disorder that is characterized by progressive cognitive impairment. Objective measures of cognitive function may provide reliable neurobiomarkers for patients with schizophrenia. The goal of the current work is to explore the correlation between resting theta power and cognitive performance in patients with schizophrenia.

**Methods:**

Twenty-two patients with schizophrenia and 23 age-, sex-, and education-matched healthy controls were included in this study. The MATRICS Consensus Cognitive Battery (MCCB) was used for cognitive evaluation and the Positive and Negative Syndrome Scale (PANSS) for evaluation of clinical symptoms. EEGs were acquired in the resting state with closed and opened eyes. Between the two groups, we compared the relative theta power and examined their relationship with cognitive performance.

**Results:**

Compared to healthy controls, patients with schizophrenia showed significantly higher theta power, both with eyes closed and open (*P* < 0.05). When the eyes were open, negative correlations were found in patients with schizophrenia between theta power in the central and parietal regions with processing speed scores, and between the theta power of the Pz electrode and verbal learning and reasoning and problem-solving scores (r ≥ −0.446). In the control group, theta power over the Fz electrode was negatively correlated with processing speed (*r* = −0.435).

**Conclusions:**

Our findings showed that theta activity increased in certain brain regions during resting state in schizophrenia. Negative associations between resting theta power (increased) over the parietal-occipital regions with MCCB domains scores (decreased) suggest that altered theta activity can be used as a neurobiological indicator to predict cognitive performance.

## Introduction

Schizophrenia is a common but severe mental disorder with a chronic duration and high disability rate that places significant pressure on patients, their families, and society as a whole (Marder and Cannon, 2019). Cognitive impairment is one of the core symptoms of schizophrenia and is directly associated with social function recovery (Mihaljević-Peleš et al., 2019; Penadés et al., 2019). Previous studies have reported that patients with schizophrenia consistently showed deficits in multiple cognitive domains, such as executive function, memory, and attention (Mihaljević-Peleš et al., 2019; Penadés et al., 2019). In addition, the severity of these defects may predict the outcome of treatment and functional recovery (Green, 2016). A series of cognitive tests have been developed that are related to the ability of identifying and processing complex tasks, such as attention, memory, learning, abstract thinking, and judgement (Roux et al., 2019; Zhang et al., 2019). The scale that is most widely used for assessing these functions is the MATRICS Consensus Cognitive Battery (MCCB) (Shi et al., 2019). However, the quantitative evaluation using MCCB often takes a long time, requires experienced psychiatrists, and the results can be affected by individual cooperation, education level, and disease status (August et al., 2012). Thus, more objective and efficient cognitive evaluation methods are needed to explore the biomarkers related to cognitive performance for schizophrenic patients.

Electrical activity in neurons is stable in stationary individuals, the complex neuronal activation and encoding thereof can be monitored and recorded by non-invasive electroencephalogram (EEG). Neural oscillations underlay the cognitive function of the brain and were considered to be biomarkers of neuropsychiatric diseases (Fries, 2005). Neurotraumatic data suggested that abnormal theta oscillations might be an early indicator of mild traumatic brain injury. Furthermore, various studies have found that resting low-frequency (delta and theta) activity increased in patients with schizophrenia (Venables et al., 2009; Garakh et al., 2015; Newson and Thiagarajan, 2018; Perrottelli et al., 2021). In remitted schizophrenia, theta band current source density over the anterior cingulate cortex, as well as the connectivity between the bilateral inferior parietal lobe or the left inferior parietal lobe and the right middle frontal gyrus has been found to be elevated (Shreekantiah Umesh et al., 2016). Furthermore, a review has recently reported that theta activity may be highly involved in memory encoding and retrieval and inhibitory control regulation, and thus played a key role in speech comprehension and other extended cognitive processes (Cavanagh and Frank, 2014). However, little is known about the relationships between resting theta power and multiple cognitive domains in patients with schizophrenia. The present study aimed to evaluate the cognitive function of schizophrenic patients using MCCB and to subsequently identify correlations with resting theta power to determine a reliable biomarker for cognitive evaluation.

## Materials and Methods

### Participants

A total of 45 individuals were recruited to participate in our study from Beijing Anding Hospital, Capital Medical University. This cohort included 22 patients with schizophrenia (SCH) and 23 health controls (HCs). The patients were validated with the Structured Clinical Interview for DSM-IV (SCID), aged 18–60 years, with education above junior middle school. HCs were recruited by advertisement and had no DSM-IV axis I disorders. All participants had no neurological disease. This study was approved by the Ethics Committee of Beijing Anding Hospital, Capital Medical University, China.

### Clinical and Neuropsychological Assessment

The clinical symptoms of each patient were evaluated with the Positive and Negative Symptom Scale (PANSS, Chinese version), which was analyzed by two factors of negative symptoms, positive symptoms. The MATRICS consensus cognitive battery (MCCB, Chinese version) was used to evaluate cognitive deficits in patients with schizophrenia and healthy controls (Shi et al., 2019). All cognitive survey scores were computerized to avoid human-factor imaging, and cognitive testing was conducted by systematic training.

Resting-state EEGs were recorded using a 128-electrode scalp cap (HydroCel Geodesic Sensor Net, Electrical Geodesics, Inc., Eugene, OR, United States). Data for eyes closed and eyes opened were collected for 5 min each. During each recording, the reference electrode was set as the default Cz, and online filtering was implemented with a bandpass filter with cutoff frequencies of 0.01 and 100 Hz. The sampling rate was 1000 Hz and the scalp impedance was maintained below 50 kΩ for all electrodes. Using the EEGLAB (Delorme and Makeig, 2004), channels containing continuous artifacts were removed and interpolated using spherical spline interpolation. The EEG data was then re-referenced to the average reference with whole brain. Any stereotypical artifacts, such as eye blinks, eye movements, and muscle tension, were separately removed using an automatic artifact rejection method based on the independent component analysis (ICA) (McLoughlin et al., 2014), which is a blind source separation algorithm. Theta band (4∼8 Hz) power of each electrode was extracted, and then the relative power in theta band was calculated. The formula to calculate relative power was as follows, where *f*_1_ is the lower frequency and *f*_2_ is the higher frequency.


$$\text{Relative}\,\text{Power}\,\left(f\,1,f\,2\right)=\frac{P\,o\,w\,e\,r\,\left[\left(f\,1,f\,2\right)\right]}{P\,o\,w\,e\,r\,\left(2,25\right)}\times 100\%$$

The scalp regions utilized in this study were in accordance with standard methods in resting-state EEG research. In the comparison between the patient group and the healthy control group, the mean of relative power in six selected regions and at four sites along the midline were regarded as the resting-state EEGs indexes based on previous literature (Clarke et al., 2001). Six brain areas were identified as follows: the left frontal lobe (F3, F7 electrodes), the right frontal lobe (F4, F8 electrodes), the left central area (T3, C3 electrodes), the right central area (T4, C4 electrodes), the left posterior brain area (T5, P3, O1 electrodes), and the right posterior brain area (T6, P4, O2 electrodes). The four sites along the midline were considered as the Fz, CPz, Pz, and Oz electrodes.

### Statistical Analysis

Statistical analysis was performed using the SPSS 23.0 software. Categorical data was analyzed by Chi-square test, and numerical data was analyzed by MANOVA. Numerical data was expressed as the mean ± standard deviation (μ ± σ). We analyzed the EEG data after standardizing the Z score. The Z score calculation formula: Z (patient) = [X (Theta power of patient group) − μ (Mean theta power of the control group)]/σ (SD of the control group). We then analyzed the Pearson correlation between theta Z score and MCCB domain scores and further conducted a partial correlation analysis on the clinically substantial variables again after controlling for factors such as sex; age, married, and education. For all of the preceding analyses, if the *P*-value is less than 0.05, it was considered to represent statistically significant differences. And *P*′ represents the corrected *P*-value after the Bonferroni correction.

## Results

### Demographics Characteristics Result

The demographic characteristics and clinical data for schizophrenia and health controls are summarized in Table 1. There were no significant differences in sex (χ^2^= 0.668, *P* = 0.767), age (*t* = 0.133, *P* = 0.190), Married (χ^2^= 0.279, *P* = 0.337), and education (*t* = 1.579, *P* = 0.122) among two groups.

**TABLE 1:** Demographic and clinical characteristics of schizophrenia and control.

Item/group	SCH *n* = 22	HCs *n* = 23	*t/*χ*^2^*	*P*
Sex (male)	10	9	0.668	0.767
Age (years)	32.00 ± 12.12	27.52 ± 10.42	0.133	0.190
Married	8	5	0.279	0.337
Education (years)	13.59 ± 2.28	14.65 ± 2.28	1.579	0.122
**Psychiatric symptoms**				
Positive symptom score of PANSS	18.36 ± 6.91	—	—	—
Negative symptom score of PANSS	16.59 ± 8.17	—	—	—
Total score of PANSS	71.00 ± 22.87	—	—	—

*SCH, schizophrenia; HCs, health controls; PANSS, Positive and Negative Syndrome Scale.*

### MATRICS Consensus Cognitive Battery Result

Compared to healthy control, the MCCB tests score in schizophrenia group was significantly lower in total score *F*_(1,43)_ = 105.02, *P* < 0.001, speed of processing *F*_(1,43)_ = 32.96, *P* < 0.001, verbal learning and memory *F*_(1,43)_ = 71.44, *P* < 0.001, working memory *F*_(1,43)_ = 65.11, *P* < 0.001, reasoning and problem solving *F*_(1,43)_ = 38.64, *P* < 0.001, visual learning and memory *F*_(1,43)_ = 28.50, *P* < 0.001, and Attention/Vigilance *F*_(1,43)_ = 26.11, *P* < 0.001. No significant difference was found between two groups in the social cognitive dimension *F*_(1,43)_ = 2.16, *P* = 0.149 (see Table 2).

**TABLE 2:** Comparison of cognitive domain between schizophrenia and control.

Cognitive domain	SCH *n* = 22	HCs *n* = 23	*F*	*P*	*P*′
Speed of processing	46.41 ± 9.26	61.26 ± 8.08	32.96	<0.001**	<0.01**
Verbal learning and memory	45.18 ± 7.84	60.96 ± 4.25	71.44	<0.001**	<0.01**
Reasoning and problem solving	43.09 ± 7.28	56.26 ± 6.93	38.64	<0.001**	<0.01**
Visual learning and memory	47.27 ± 7.32	56.79 ± 4.32	28.50	<0.001**	<0.01**
Working memory	46.59 ± 10.06	65.78 ± 5.26	65.11	<0.001**	<0.01**
Attention/vigilance	42.27 ± 9.83	54.96 ± 6.57	26.11	<0.001**	<0.01**
Social cognition	49.91 ± 9.69	53.74 ± 7.71	2.16	0.149	>0.1
MCCB total score	44.41 ± 6.28	61.00 ± 4.47	105.02	<0.001**	<0.01**

**P < 0.01.

### Theta Power Result

**FIGURE 1:**
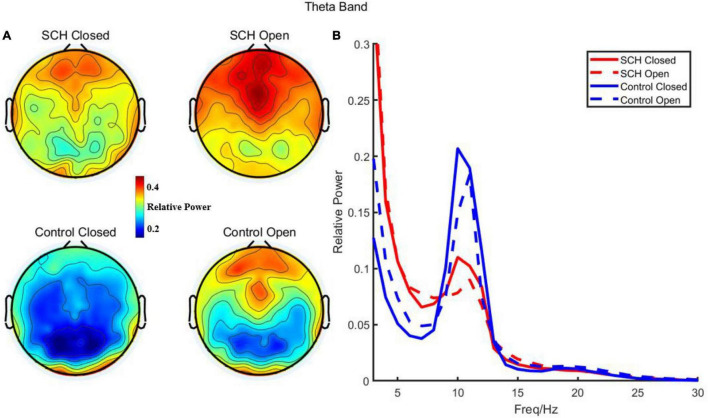
**(A)** Relative power of theta in schizophrenia and control with eye closed and eye open. **(B)** Relative power and frequency in schizophrenia and control with eye closed and eye open (channel 72).

**FIGURE 2:**
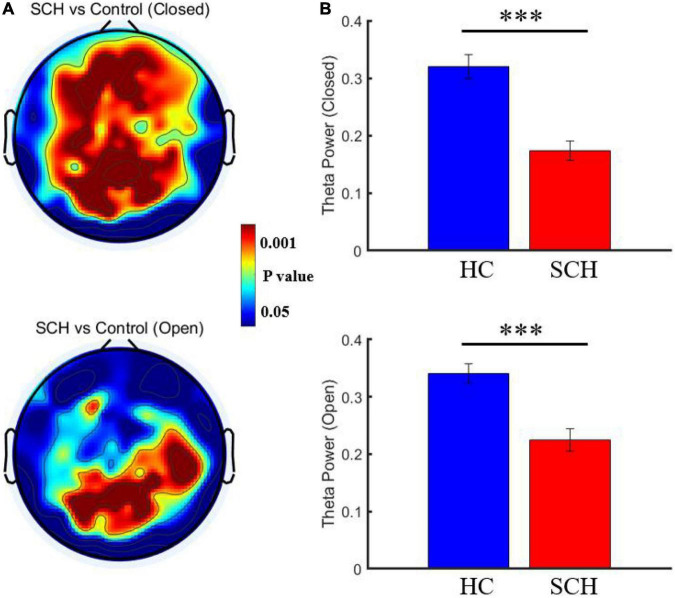
**(A)** Comparison of resting theta power between schizophrenia and control with eye closed and eye open. **(B)** Comparison of the mean theta power from channel 72 between schizophrenia and control with eye closed [*P* = 1.9073*10^(−6)^] and eye open [*P* = 2.9624*10^(−5)^]. ***P < 0.0001.

Compared to healthy controls, schizophrenia exhibited significantly higher theta power with eyes closed and eyes open (*P* < 0.05) (Figures 1, 2). Specifically, we selected six selected regions and four sites based on previous literature. We analyzed the group differences between schizophrenia and control with eyes closed and eyes open, respectively (see Tables 3, 4). There were significant differences with Bonferroni correction (*P′* ≤ 0.03) in theta power between the two groups in all regions and midline sites with eyes closed. There were also significant differences in theta power between two groups in all regions and midline sites with eyes open but only right central [*F*_(1,43)_ = 14.309, *P* < 0.001, *P*′ < 0.01], right posterior[*F*_(1,43)_ = 14.183, *P* < 0.001, *P*′ < 0.01], and Pz [*F*_(1,43)_ = 11.878, *P* = 0.001, *P′* = 0.01] remained significant after Bonferroni correction.

**TABLE 3:** Comparison of theta power between schizophrenia and controls with eyes closed.

Brain areas	SCH *n* = 22	HCs *n* = 23	*F*	*P*	*P*′
Left frontal	0.358 ± 0.115	0.232 ± 0.080	18.182	<0.001**	<0.01**
Left central	0.336 ± 0.134	0.217 ± 0.083	14.464	<0.001**	<0.01**
Left posterior	0.310 ± 0.109	0.194 ± 0.075	17.814	<0.001**	<0.01**
Right frontal	0.360 ± 0.114	0.242 ± 0.088	15.417	<0.001**	<0.01**
Right central	0.342 ± 0.123	0.247 ± 0.069	10.266	0.003**	0.03*
Right posterior	0.322 ± 0.106	0.194 ± 0.082	20.731	<0.001**	<0.01**
Fz	0.250 ± 0.095	0.399 ± 0.124	20.766	<0.001**	<0.01**
CPz	0.366 ± 0.109	0.254 ± 0.091	14.007	0.001**	0.01*
Pz	0.337 ± 0.1095	0.193 ± 0.073	26.831	<0.001**	<0.01**
Oz	0.294 ± 0.119	0.186 ± 0.106	10.275	0.003**	0.03*

*P < 0.05, **P < 0.01.

**TABLE 4:** Comparison of theta power between schizophrenia and controls with eyes open.

Brain areas	SCH *n* = 22	HCs *n* = 23	*F*	*P*	*P*′
Left frontal	0.402 ± 0.106	0.334 ± 0.062	6.988	0.011*	0.11
Left central	0.363 ± 0.127	0.289 ± 0.090	5.140	0.028*	0.28
Left posterior	0.333 ± 0.103	0.247 ± 0.095	8.542	0.006**	0.06
Right frontal	0.395 ± 0.103	0.327 ± 0.063	7.392	0.009**	0.09
Right central	0.375 ± 0.103	0.276 ± 0.072	14.309	0.001**	0.01*
Right posterior	0.346 ± 0.095	0.242 ± 0.091	14.183	0.001**	0.01*
Fz	0.385 ± 0.071	0.443 ± 0.110	4.518	0.039*	0.39
CPz	0.394 ± 0.103	0.330 ± 0.092	4.786	0.034*	0.34
Pz	0.357 ± 0.100	0.257 ± 0.094	11.878	0.001**	0.01*
Oz	0.320 ± 0.098	0.231 ± 0.107	8.317	0.006**	0.06

*P < 0.05, **P < 0.01.

### Correlation Result

**FIGURE 3:**
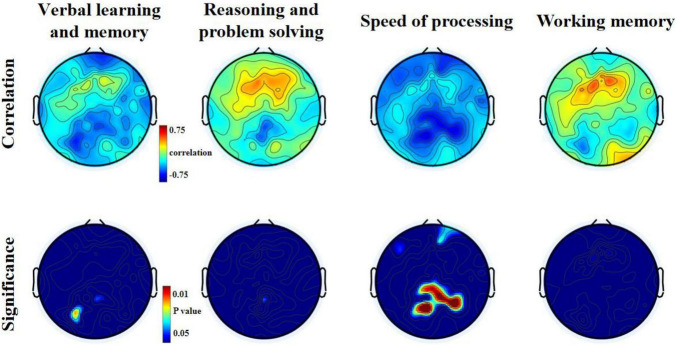
Relationship between theta relative power and MCCB domains T-scores for the eyes open condition in patients with schizophrenia.

With open eyes in schizophrenia, we found significant correlations in four dimensions of MCCB score. In verbal learning and memory score, relative theta power was found to be negatively correlated to six electrodes (*p* < 0.05). In verbal learning and memory score, relative theta power was found to be negatively correlated to six electrodes (*p* < 0.05), locating in the left posterior region. In reasoning and problem-solving score and working memory score, two electrodes (*p* < 0.05) showed negative significant correlation to relative theta power. In speed of processing score, 32 electrodes (*p* < 0.05, *p*-value for 10 of them is <0.01) showed negative significant correlation to relative theta power, locating in parietal-occipital region and right frontal region (Figure 3 and see Tables 5–8 for detailed data in specific region or electrodes). However, we did not find a correlation between cognition scores and relative theta power in schizophrenia and control with eyes closed.

**TABLE 5:** Relationship between theta relative power and MCCB domains scores with eyes open in schizophrenia.

Cognitive domain	Brain area									
	Fz	CPz	Pz	Oz	Left frontal	Left central	Left posterior	Right frontal	Right central	Right posterior
Speed of processing	−0.316	−0.550**	−0.479*	−0.379	−0.303	−0.282	−0.393	−0.291	−0.371	−0.460*
Verbal learning and memory	−0.100	−0.365	−0.446*	−0.346	−0.084	−0.132	−0.345	−0.183	−0.291	−0.248
Reasoning and problem solving	0.245	−0.205	−0.461*	−0.255	0.197	−0.015	−0.052	0.158	−0.097	−0.103
Visual learning and memory	0.397	0.232	0.028	0.093	0.316	0.197	0.050	0.318	0.197	0.123
Working memory	0.258	0.003	−0.109	−0.119	0.157	0.118	−0.149	0.064	−0.093	−0.111
Attention/vigilance	0.208	0.048	−0.145	−0.225	0.066	0.036	−0.089	0.008	−0.095	−0.028

*Correlation is significant at P < 0.05 level; **Correlation is significant at P < 0.01 level.

**TABLE 6:** Relationship between theta relative power and MCCB domains scores with eyes open in control.

Cognitive domain	Brain area									
	Fz	CPz	Pz	Oz	Left frontal	Left central	Left posterior	Right frontal	Right central	Right posterior
Speed of processing	−0.435*	−0.035	0.146	0.174	−0.052	0.184	0.176	0.049	0.246	0.159
Verbal learning and memory	−0.047	0.039	−0.087	0.052	−0.132	−0.046	0.014	0.068	−0.060	−0.083
Reasoning and problem solving	−0.001	−0.088	−0.095	−0.085	−0.064	−0.036	−0.021	−0.065	−0.036	−0.058
Visual learning and memory	0.015	−0.251	0.112	0.087	−0.107	0.173	0.061	−0.168	0.268	0.211
Working memory	−0.009	−0.069	0.027	0.239	0.123	0.260	0.247	0.287	0.287	0.221
Attention/vigilance	−0.152	−0.178	−0.051	0.101	−0.077	0.088	0.106	0.056	0.060	−0.028

*Correlation is significant at P < 0.05 level.

**TABLE 7:** Relationship between theta relative power Z-scores and MCCB domains T-scores for the eyes open condition in patients with schizophrenia.

Cognitive domain	Brain area									
	Z-Fz	Z-CPz	Z-Pz	Z-Oz	Z-Left frontal	Z-Left central	Z-Left posterior	Z-Right frontal	Z-Right central	Z-Right posterior
Speed of processing	−0.316	−0.550**	−0.479*	−0.379	−0.303	−0.282	−0.393	−0.291	−0.371	−0.460*
Verbal learning and memory	−0.100	−0.365	−0.446*	−0.346	−0.084	−0.132	−0.345	−0.183	−0.291	−0.248
Reasoning and problem solving	0.245	−0.205	−0.461*	−0.255	0.197	−0.015	−0.052	0.158	−0.097	−0.103
Visual learning and memory	0.397	0.232	0.028	0.093	0.316	0.197	0.050	0.318	0.197	0.123
Working memory	0.258	0.003	−0.109	−0.119	0.157	0.118	−0.149	0.064	−0.093	−0.111
Attention/vigilance	0.208	0.048	−0.145	−0.225	0.066	0.036	−0.089	0.008	−0.095	−0.028

*Correlation is significant at P < 0.05 level; **Correlation is significant at P < 0.01 level. Similar to previous results, the results of partial correlation analysis after controlling sex; age, married, and education.

**TABLE 8:** Partial correlation between cognitive domain and brain area.

Cognitive domain	Brain area		
	CPz	Pz	Right posterior
Speed of processing	−0.628**	−0.496*	−0.560*
Verbal learning and memory	0.247	0.068	0.056
Reasoning and problem solving	−0.394	−0.608**	−0.297

*P < 0.05, **P < 0.01.

## Discussion

We found that in open-eye state, the theta power was significantly higher than when eyes were closed in patients with schizophrenia. The resting theta power across the brain in the patient group was also significantly higher in amplitude than the control group. Our correlation analysis indicated that increased theta power in the frontal midline electrode exhibited a significant relationship with lower working memory and processing speed scores in healthy controls. In patients with schizophrenia, an increase in theta power in the occipital midline electrode led to significantly reduced verbal learning and reasoning and problem-solving scores. Furthermore, an increase in theta power in the midline and right posterior brain regions was significantly related to lower processing speed scores.

Previous studies have found that there were apparent changes in the EEG from the state of closed eyes to open eyes. These changes were usually treated as reflection of brain activity reorganization in reply to visual perception (Barry et al., 2007). Previous studies in schizophrenia have also found different patterns of frequency band changes between patients with eyes open and eyes closed (Newson and Thiagarajan, 2018). Besides, most rest EEG studies in schizophrenia have been conducted during eyes closed, when alpha oscillations are strongest, whereas only a handful of studies have investigated rest EEG in schizophrenia during eyes open, when theta rhythms are most easily observed (Zhang et al., 2021). Therefore, we included the rest data with eyes open and eyes closed for analysis.

Evidence from previous EEG research has reported that in patients with schizophrenia during the eyes closed condition, resting delta and theta power are consistently and reliably increased, with a consistency score of 2.2 and a reliability score of 1.446. By contrast, alpha power in these patients decreased compared to healthy controls. The largest difference was reported as an average increase of 50% in theta and an average decrease of 58% in alpha, and were also noted in schizophrenia patients compared to those with other psychiatric disorders (Phillips and Uhlhaas, 2015; Newson and Thiagarajan, 2018). These results fell in line with our results in that we also observed significantly increased resting theta power and decreased alpha power across the brain in schizophrenic patients compared to healthy controls. A study by Venables et al. suggested that resting low-frequency (delta and theta) activity increased and correlated with catechol-O-methyltransferase (COMT) 158Met genetic polymorphism in patients with schizophrenia. However, no similar low-frequency abnormalities were found in patients with bipolar disorder or their relatives, as well as relatives of schizophrenic patients (Venables et al., 2009). The authors speculated that characteristic pathological features of schizophrenia and the effects of COMT 158Met polymorphism on dopaminergic neuronal function may contribute to the abnormal low-frequency resting EEGs. The current hypothesis about the effect of COMT on the dopaminergic activity of the nucleus accumbens and prefrontal cortex proposes that Met allele may lead to an increase in tonic dopamine and reduction in phasic dopamine, increasing the likelihood of negative symptoms and pathological discomfort (Bilder et al., 2004). Furthermore, other studies have found that healthy brain function is closely associated with increased resting alpha power and reduced theta power. Similar changes in alpha and theta power can be observed between children and adults, while the converse changes occur during progressive aging. Overall, compared with age-matched controls, patients with a neuropsychiatric illness have increased theta power and decreased alpha power. Some researchers believe that the impaired ability to respond to external stimuli is often accompanied with increase in theta power and a decrease in alpha power.

In the correlation analysis, we found that resting theta power over the frontal midline region for the eyes-open condition was negatively correlated with processing speed and working memory scores in the control group. A neuroimaging study showed that in healthy controls correct working memory retrieval was associated with activation in the bilateral fronto-parieto-occipital network, which spans across the dorsolateral and ventrolateral prefrontal cortex, as well as the superior parietal cortex (Koch et al., 2008). Cognitive performance could be reflected by theta activity, which plays a central role in memory encoding and inhibitory control modulation (Headley and Paré, 2017). Moreover, theta power may provide an envelope for widespread integration of cortical areas participating in language and other extended cognitive processes (Halgren et al., 2015). In the resting state, theta activation may result in insufficient output on tasks and reduced task-related theta power, indicating an association between increased theta activity and deficits in cognitive function. A neurodevelopmental study conducted by Rodriguez-Martinez et al. (2013) reported that in the brain maturation of children, the theta power decreased and working memory scores increased with age. In addition, the critical role of theta oscillations in neuronal information processing was also noted, particularly in the hippocampus (Stella and Treves, 2011). Along these same lines, in patients with multiple sclerosis, frontal theta levels can be considered an inverse marker of processing speed (Keune et al., 2019).

Our findings from the correlation analysis showed that resting theta power over the midline had a negative relationship with verbal learning, processing speed, reasoning and problem-solving scores in patients with schizophrenia. We also found that the scores of verbal learning and memory were negatively correlated with scores of PANSS positive symptoms. It is known that patients with schizophrenia have verbal memory deficits before disease onset, which are considered one of the most sensitive predictors of schizophrenia (Woodberry et al., 2013). During the remittal phase of schizophrenia, theta connectivity consistently increases between the anterior cingulate cortex and bilateral inferior parietal lobe, and between the left inferior parietal lobe and the right middle frontal gyrus (Shreekantiah Umesh et al., 2016). Functional connectivity results indicate an abnormal increase in resting theta band functional connectivity across the midline, sensorimotor, orbitofrontal regions and the left temporoparietal junction, which may mediate verbal memory defects in schizophrenic patients and high-risk individuals (Andreou et al., 2015). The authors of this study believed that increased theta-band connectivity in the resting state may be related to the over-activation of the default mode network, which can lead to memory defects due to an inappropriate modulation of theta band activity during memory retrieval episodes. Disturbed theta oscillations may also cause impairment of working memory under theta-gamma coupling effects in patients with schizophrenia (Halgren et al., 2015). Krukow et al. performed a functional connectivity analysis of 32 patients with first-episode schizophrenia using resting-state EEG and found a low theta band phase lag index (PLI) in the left frontal cortex and a high PLI within the somatosensory cortex. This work also suggested that the duration of untreated disease helped to predict deficits in the processing speed of patients, and indicated that abnormal cortico-cortical synchronization contributes to cognitive slowing in schizophrenia (Krukow et al., 2018). A meta-analysis also revealed that schizophrenic patients with higher brain-derived neurotrophic factor levels performed better on reasoning/problem solving tasks. Similarly, another meta-analysis found that patients with schizophrenia perform better in reasoning/problem-solving tasks when brain-derived neurotrophic factor levels are higher (Ahmed et al., 2015), and that this level decreased when the cerebellar theta burst continued to be stimulated (Sanna et al., 2020).

### Limitations

There are some limitations of this study that should be mentioned. In this study, we included a relatively small sample size, which resulted in a loss of statistical power. The type and dosage of medication were also not recorded in detail, which may affect the results of this work. Furthermore, multiple frequency bands were not analyzed, and information in oscillations other than theta may play a role in this disease. Further research with a larger sample size with matched controls is required in the future to find more neurocognitive-related neurobiological markers.

Overall, on the basis of our previous studies and current work, we analyzed the correlations between resting theta power and MCCB domains scores. We primarily found negative relationships between theta power and the scores of cognitive domains such as verbal learning memory, processing speed, reasoning, and problem solving. These results suggest that theta power may be a neurobiological marker that quantitatively reflects cognitive performance.

## Data Availability Statement

The original contributions presented in the study are included in the article/supplementary material, further inquiries can be directed to the corresponding authors.

## Ethics Statement

The studies involving human participants were reviewed and approved by Ethics Committee of Beijing Anding Hospital (ID: 201723SF-2), Capital Medical University. The patients/participants provided their written informed consent to participate in this study.

## Author Contributions

XZ, YC, CH, and SS conceived and designed the study. XZ and YC contributed to the literature search. CH, XP, ZS, GL, YX, YZ, JL, PZ, and WD contributed to data collection, data analysis, and the interpretation of results. All authors contributed to writing the manuscript and approved the submitted version.

## Conflict of Interest

MG was employed by WM Therapeutics Ltd. The remaining authors declare that the research was conducted in the absence of any commercial or financial relationships that could be construed as a potential conflict of interest.

## Publisher’s Note

All claims expressed in this article are solely those of the authors and do not necessarily represent those of their affiliated organizations, or those of the publisher, the editors and the reviewers. Any product that may be evaluated in this article, or claim that may be made by its manufacturer, is not guaranteed or endorsed by the publisher.
